# Exploring Students’ Perceptions and Usage of Artificial Intelligence in Supporting Mental Health: A Preliminary Study in Higher Education in Qatar

**DOI:** 10.3390/healthcare14091247

**Published:** 2026-05-06

**Authors:** Amani Safwat ElBarazi, Hatem Mohamed, Ramzi Nasser

**Affiliations:** College of Education and Arts, Lusail University, Doha P.O. Box 9717, Qatar; hmohamed@lu.edu.qa (H.M.); rnasser@lu.edu.qa (R.N.)

**Keywords:** artificial intelligence, trust, usage, mental health support, privacy

## Abstract

**Highlights:**

This study is one of the first empirical studies investigating AI-assisted mental health perceptions among university students in the Arab Gulf region.

**What are the main findings?**

- University students demonstrated moderate awareness and cautious acceptance of AI in mental health, with trust emerging as a significant predictor of readiness to use AI-based tools.
- Key concerns included privacy, diagnostic accuracy, and limited emotional empathy, while students showed a clear preference for AI as a complementary rather than a re-placement tool.

**What are the implications of the main findings?**

- Findings support the implementation of hybrid mental health care models in universities, where AI enhances early screening, accessibility, and continuous support along-side human counseling.
- Institutional adoption of AI should be guided by robust ethical frameworks, emphasizing data privacy, transparency, cultural sensitivity, and digital mental health literacy.

**Abstract:**

Background: Artificial intelligence (AI) is widely used in mental health care for screening, monitoring, and intervention. Notably, most studies of AI in mental health have been performed in Western contexts, with limited evidence from the Arab Gulf region, where cultural factors such as stigma, privacy, and help-seeking norms may influence acceptance. Objective: Investigating university students’ perceptions of AI in mental health support, including awareness, trust, readiness, and preferences in a Gulf context. Methods: A cross-sectional survey was administered to 220 university students in Qatar. Data were analyzed using descriptive statistics, Chi-square tests, and one-way ANOVA to explore associations and group differences. Results: Students showed low-to-moderate levels of awareness and trust in AI-based mental health tools. The majority of participants showed that they were prepared to employ AI for stress management, but they do not prefer to replace face-to-face therapy, suggesting a preference for complementary use. A significant association was found between readiness and expectations (*p* < 0.00001), which means ambivalence toward AI effectiveness. No significant differences were observed across gender or academic level (*p* > 0.05). Key concerns included loss of human interaction, overreliance on technology, and diagnostic accuracy, while perceived benefits included cost reduction and 24/7 accessibility. Conclusions: Students exhibit cautious adoption of AI in mental health services. Acceptance is influenced by trust, privacy issues, and apparent compassion. AI is optimally situated as a supplementary instrument within ethically regulated, culturally attuned hybrid care frameworks that maintain the fundamental importance of human connection.

## 1. Introduction

Artificial intelligence (AI) is increasingly integrated into mental health care as a tool to support screening, monitoring, and intervention [1,2]. The rise of anxiety and depression among college students stems from the ever-increasing demands and responsibilities among young adults around the world, especially university students [3]. AI-driven approaches suggest a scalable and innovative complement to traditional mental health services, enhancing accessibility and efficiency while preserving the central role of human therapists in delivering empathetic psychological care [4,5,6]. AI applications in mental health include emotional monitoring, early detection, digital interventions, and personalized support systems [7,8,9]. The use of new, algorithmic, ever-adapting learning models has surged to diagnose psychiatric symptoms and behavioral patterns. These tools are equipped with conversational agents and chatbot-based cognitive–behavioral therapies easily accessible on the internet [10,11,12]. Recent research in AI, including natural language processing and affective computing, has enhanced the ability of digital systems to detect psychological patterns and deliver personalized mental health support [5,13]. In addition, some AI-driven platforms integrate data from wearable devices, such as heart-rate variability, sleep patterns, and activity levels, to provide real-time feedback and support [14,15]. While these findings expand the scope of AI in mental health care, their effectiveness and adoption depend largely on readiness to engage with such technological tools, particularly among university students [16].

University students around the world are extensively using digital tools to support their learning [17,18]. While university counseling centers remain active, students are increasingly using AI-assisted mental health tools [19,20]. However, several important concerns remain regarding the use of AI in mental health. Students are concerned about privacy, expected effectiveness, and authenticity in the use of AI as a digital mental health “companion” [21,22]. Some researchers have underlined the importance of implementing AI within diverse educational settings to support young people who are vulnerable to mental issues [23,24].

In the Arab world, sociocultural norms play a significant role in shaping attitudes toward mental health and help-seeking behaviors. Empirical studies have consistently shown that stigma, fear of social judgment, and concerns about reputation act as major barriers to accessing formal mental health services [25,26]. As a result, individuals often prefer anonymity and confidentiality and may avoid face-to-face counseling due to embarrassment or fear of being labeled, leading them to seek more covert or indirect forms of support [27,28]. In this context, AI-driven mental health technologies may offer a more acceptable alternative by reducing perceived stigma and enabling private access to support. However, despite these advantages, concerns remain regarding the lack of empathy, emotional understanding, and human connection in AI-based systems [29].

AI-powered technologies, including mobile applications, support medication adherence, mood tracking, and identification of emotional triggers [30,31]. Additionally, digital phenotyping uses behavioral data derived from online activities to infer and evaluate mental well-being [32,33,34,35,36]. Ethical issues such as data privacy, regulatory constraints, and access to diverse datasets complicate the utilization of these technologies [37,38].

Individuals are more likely to engage with AI systems that are perceived as reliable, trustworthy, and safe for emotional support [39]. While people are more likely to provide sensitive information and feelings about their personal lives to AI chatbots than they would to face-to-face human therapists, out of fear of disclosure and shaming [40,41]. While AI-based tools could diminish psychological barriers to seeking assistance, they also provoke inquiries regarding accountability, diagnostic accuracy, empathy, and ethical practice [42].

A recent study has shown that students’ willingness to utilize AI in mental health is associated with their mental health level. Students who experienced higher levels of depressive or anxiety symptoms showed greater openness toward using AI in mental health support. Those students may view AI tools as helpful alternatives when traditional human therapies are less accessible. In addition, students tend to accept AI more when it complements human therapy in a blended care rather than replacing the therapeutic relationship entirely [43]. Recent research suggests relatively consistent levels of technological familiarity and engagement across gender and academic levels within similar educational contexts among university students [44]. This convergence in digital usage may result in comparable perceptions of AI-based mental health tools across demographic groups [45].

Understanding the adoption of AI in mental health necessitates an analysis of certain psychological and behavioral factors that influence users’ perceptions and interactions with these technologies. Previous studies in digital mental health and technology acceptance identify various dimensions, including awareness, trust, readiness, expectations, and perceived concerns [46].

First, awareness can be described as individuals’ knowledge and familiarity with AI applications in mental health. Studies have shown that higher awareness is associated with greater openness to employing AI in mental health because people are more likely to understand its potential benefits and limitations [47,48].

Second, trust is a critical factor influencing AI adoption in mental health. Trust involves assessments of reliability, confidentiality, accuracy, and emotional appropriateness of AI innovations. Prior studies demonstrate that insufficient trust is a significant barrier to the adoption of AI in mental health. Most concerns are related to privacy and empathy concerns [5,49].

Third, readiness to use AI in mental health can be described as behavioral intention and reflects the extent to which individuals are ready to engage with AI-based tools in real-world contexts. Readiness is influenced by both cognitive evaluations (e.g., perceived benefits) and affective responses (e.g., comfort), consistent with technology acceptance frameworks [22]. In addition, expectations regarding the effectiveness and ease of using AI in providing psychological support play a critical role in shaping attitudes and intentions [50]. Users who believe that AI can provide helpful psychological support are more likely to consider its benefits, although this does not always translate into actual behavioral readiness [38].

Finally, perceived concerns, including privacy risks, lack of empathy, and diagnostic accuracy, represent important barriers that may decrease the probability of using AI in obtaining psychological counseling despite perceived benefits [51]. These concerns are significantly relevant in mental health settings in which trust, privacy, and emotional understanding are essential [52].

The present study is grounded in established models of technology acceptance, including the Technology Acceptance Model (TAM) [53,54,55,56] and the Unified Theory of Acceptance and Use of Technology (UTAUT) [57], which emphasizes the role of cognitive, emotional, and behavioral factors in shaping users’ perspectives of using technologies.

Building on these frameworks, the current study proposes a conceptual model that organizes students’ perceptions of AI in mental health into three interrelated domains. First, cognitive–perceptual factors, including awareness and expectations, reflect students’ knowledge and beliefs about AI benefits. Second, affective–evaluative factors, including trust and concerns that focus on emotional and evaluative responses toward AI. Affective–evaluative factors are connected to reliability, privacy, and empathy. Third, behavioral intention factors, including readiness and preference, represent students’ readiness to engage with AI-based tools and their comparative evaluation of AI versus traditional face-to-face counseling.

Within this model, awareness and expectations are proposed to influence trust, while trust and concerns together impact readiness to use AI technologies. Preference is conceptualized as an outcome reflecting students’ evaluation of AI as a complement or an alternative to human-delivered mental health services. This model provides a structured approach to understanding how university students evaluate and adopt AI-based mental health tools within a culturally specific context.

Despite growing global interest in AI-based mental health interventions, most research has been conducted in Western contexts, with limited attention to the Arab Gulf region. Existing studies in the Middle East remain scarce and often focus on general digital health adoption rather than users’ psychological perceptions and trust in AI systems [58,59]. Addressing this gap, the present study examines university students’ perceptions, trust, and readiness to use AI in mental health support within a Gulf context [60].

**Research Questions**

Despite the growing adoption of AI in mental health care, important questions remain regarding how students perceive and engage with these technologies. Accordingly, this study addresses the following research questions:

Question 1: To what extent do university students demonstrate awareness of, trust, readiness, and preference for AI-based tools for mental health support

Question 2: How ready and willing are students to use AI-powered stress management tools to improve their mental health?

Question 3: Do gender and academic levels have a large effect on how much students know about AI in mental health, how much they trust it, how ready they are for it, or how apt they are to use it?

Question 4: What are the main concerns and perceived benefits of students regarding the incorporation of AI into university mental health services?

**Research Hypotheses**

**H1.**  *University students will report low-to-moderate levels of awareness and trust in using AI in mental health support.*

**H2.**  *University students will demonstrate a moderate level of readiness and preference for using AI technologies in mental health support.*

**H3.**  *There is a strong positive relationship between being ready and expecting neurocognitive AI-assisted mental health support (expectations).*

**H4.**  *There will be no statistically significant differences in students’ awareness, trust, readiness, or preferences regarding AI-based mental health tools based on gender or academic level.*

## 2. Materials and Methods

### 2.1. Study Design

The study employs a cross-sectional design. This study adhered to the STROBE (Strengthening the Reporting of Observational Studies in Epidemiology) principles of research write-up. This study explores university students’ perceptions and usage of AI in supporting mental health. This study’s survey allowed for the efficient collection of data from relatively large samples of students within a limited timeframe.

### 2.2. Time and Place of Study

This study was conducted between December 2025 and January 2026 in Doha, Qatar.

### 2.3. Participants

All participants in the study were university students from an Arab Gulf University who voluntarily participated in this study. The university’s students were recruited via the university website; we announced an ad through the university’s website for students to voluntarily participate in the research. While this was effective in obtaining people’s responses, however, some limitations of self-selection bias abounds when people choose whether to take part in a study instead of being chosen at random. To address this, we carefully examined statistically significant differences within the sample and employed non-parametric tests, which are less sensitive to distributional assumptions and potential sampling imbalances. The inclusion criteria were as follows: being an active student at the university. Exclusion criteria: Students who did not enroll or register at the university were excluded from the study.

### 2.4. Research Instrument and Validation Process

The survey used in this study was designed as a structured, self-administered questionnaire to examine university students’ awareness, trust, readiness, and preferences for employing AI in mental and neurocognitive health support. The survey consisted of closed-ended and Likert-scale items, structured into five sections: (1) Demographic data such as age, gender, academic level, and faculty, (2) Prior engagement with AI applications to gain mental health assistance, (3) Awareness, trust, readiness, and preferences of employing AI in mental and neurocognitive health support. The dimensions used in the study were all made up of single-item questions and responses, with the exception of the “concern” construct. For instance, for the question of “trust”: “How much do you trust the use of AI programs for psychological treatment?” where students respond: “Very Low”, “Low”, “Moderate”, “High”, and “Very High.” The question for awareness: “What is the extent of your knowledge of AI applications in the field of mental health?” With responses of “Weak,” “Moderate,” “Good,” and “Excellent.” Table 1 presents the dimensions of the questions and response types used in the questionnaire.

### 2.5. Development and Validation by Experts

An extensive and in-depth literature review regarding AI applications in mental health was conducted. Then, the primary version of the survey was designed. The survey was created based on two broad themes of positive and negative valence, covering digital therapy adoption and technology acceptance frameworks [46,47,61,62,63,64,65,66,67,68,69,70]. The survey’s conceptual foundation draws on integrated technology acceptance, with two overarching positive valences: awareness, trust, readiness, preference, expectations, and benefits. In the context of attitudes toward AI in mental health, positive valence refers to the degree to which a person views AI as beneficial, trustworthy, effective, and attractive [71]. For example, performance expectancy of positive valence refers to the conviction that AI would improve mental health and well-being [11,12,22]. Negative valence (reversed) revolves around the concern with AI use, emphasizing the role of cognitive and emotional affect. The positive valence in an AI model can be described as the belief that a positive psychological disposition towards an object, notion, or behavior [72].

The survey was sent to 8 specialists and experts in clinical psychology, counseling, neurology, and mental health researchers in the Arab World to ensure the instrument’s content validity. The experts assessed the survey’s clarity, relevance, cultural and religious appropriateness, and alignment of the items with the study’s aims.

The survey was validated by expert evaluation following the incorporation of their feedback and comments, which included adding or deleting some items and questions. In addition, the experts’ feedback includes the rewarding of ambiguous items, simplification of terminology for enhanced understanding, and deletion of redundant questions (refer to Table 2). A Content Validity Index (CVI) was computed by summarizing the levels of agreement among experts. The CVI is a number used to measure the validity of the content of a tool or a questionnaire. A panel of experts rates how relevant, clear, and indicative the items on a tool are of the construct being measured [73]. The survey received expert validation and a CVI of 0.95. Experts decided that the survey’s items exhibited high relevance and representativeness of the intended constructions. Minor modifications were made prior to the final administration of the survey to improve conceptual clarity and cultural sensitivity in the context of Qatari universities.

Several survey items were revised following expert evaluation to enhance clarity, cultural sensitivity, and construct validity. Examples include: First, awareness (Clarity improvement): the original question was: “What is your knowledge of AI in mental health?” The revised question was: “What is the extent of your knowledge of AI applications in the field of mental health?”, which clarified the wording and aligned with measurable response categories (weak to excellent). Second, Trust (Construct precision): the original question was: “Do you trust AI for therapy?” Revised: “How confident are you in using AI for psychotherapy?” shifted from vague “trust” to measurable trust, improving alignment with the trust construct. Third, Readiness (Behavioral intention clarity): the original question was: “Would you use AI for stress?” We conceptualized readiness to the extent to which AI applications are perceived to be safe, effective, ethical, and useful within mental health services [74]. Thus, it was revised: “Are you willing to use AI applications to manage stress?” improved clarity and alignment. Fourth, preference (comparative framing): the original question was: “Do you like AI counseling?” The revised: “Would you prefer using AI applications for mental health instead of traditional face-to-face counseling?” added a comparison to human therapy for a more meaningful interpretation.

### 2.6. Pilot Testing and Reliability

After experts’ validation of the survey, pilot testing with a small group of students (*n* = 20) was conducted using the survey. The pilot test was aimed at evaluating clarity, comprehensibility, and response reliability. Students’ feedback indicated clear item interpretation and appropriate response length. To assess the internal consistency of the survey instrument, reliability analysis was conducted using Cronbach’s alpha coefficients based on the pilot sample (*n* = 20). The results indicated acceptable to good reliability across the main constructs: awareness (α = 0.82), trust (*α* = 0.86), readiness (*α* = 0.84), and preferences (*α* = 0.80). These values suggest that the survey items demonstrated satisfactory internal consistency and were appropriate for use in the main study. The completed survey demonstrated strong face and content validity, which proves its suitability for larger data collection.

### 2.7. Procedure

The university’s students were recruited via the university website; we announced an ad through the university’s website for students to voluntarily participate in the research. Students were informed that participation was voluntary, and informed consent was acquired from all participants prior to the completion of the survey. Then, students who agreed to participate in the study were asked to scan a barcode of the online version of the survey. Each student needed approximately 15–20 min to complete the survey. Confidentiality and anonymity were guaranteed throughout the research.

### 2.8. Sample Size

Sample power was used to identify an adequate sample size to detect meaningful differences in primary outcomes. A two-tailed significance test with the desired power of 0.80. A power of 0.80 (80%) means that if there is a real difference or effect in the population, the sample had an 80% chance of finding it. The parameters used for the analysis were informed by estimates drawn from previous research.

### 2.9. Ethical Considerations

This study was conducted in accordance with the ethical standards outlined in the Declaration of Helsinki (2013). Ethical approval was obtained from the Institutional Review Board (IRB) of Lusail University, Qatar (Protocol No. LU/IRB/03-12/01; approved on 3 December 2025).

All participants were provided with detailed information about the study, including its purpose, procedures, potential risks and benefits, and their rights as participants. Informed consent was obtained electronically prior to participation, and students were clearly informed that their participation was voluntary and that they could withdraw from this study at any time without consequence.

To ensure confidentiality and anonymity, no personally identifiable information was linked to participants’ responses. Data was collected through an online survey platform and stored securely in password-protected electronic systems accessible only to the research team. All data was used exclusively for research purposes, and appropriate measures were taken to protect participants’ privacy and prevent unauthorized access. No identifying information was included in any reports or publications resulting from this study.

### 2.10. Data Analysis

Descriptive statistics (means, standard deviations, frequencies, percentages) were employed to describe the sociodemographic characteristics of the sample, as well as non-parametric and parametric tests, which included Chi-Square and ANOVA tests, to examine potential differences in students’ perceptions and AI usage in mental health. The use of the Chi-square was used to address categorical data differences. The use of ANOVA rather than running multiple tests increases the chance of a false positive (inflated Type I error). Thus, ANOVA performs one overall test, keeping the error rate under control. All data were analyzed using SPSS Version 26.0, with a significance level set at *p* < 0.05.

## 3. Results

The demographic information is presented in Table 3. There was a total of 220 students who responded to the questionnaire. The majority of whom were female (78.2%) and undergraduate students (92.27%). Most participants were enrolled in the Counseling and Mental Health faculty (65.91%), followed by Business (11.82%), Information Systems (10.45%), and Law (10.00%). A smaller number were postgraduate program students (7.27%) or from English language teaching (1.82%). These figures indicate that the sample primarily represented undergraduate Counseling and Mental Health students, with females’ representation forming a significant majority of the study population.

### 3.1. Awareness and Trust

To address the first hypothesis, we looked at two questions about students’ awareness and trust in AI. With the awareness question: “How familiar are you with AI applications in mental health?” The responses to the question were 34.5% “low,” 47.7% “intermediate,” and 17.7% “high.” There was no significant difference with a χ^2^(2, *n* = 220) = 29.85, *p* < 0.0001. Thus, they were more likely to have intermediate-level knowledge-awareness of AI tools in mental health therapy. In terms of trust, we looked at one main question: “How confident are you in using AI for psychotherapy? The responses were 43.6% “low,” 47.7% “intermediate,” and 8.6% “good,” with a significant difference between the response categories with χ^2^(2, *n* = 220) = 60.9, *p* < 0.0001. Most respondents have low-to-intermediate trust in an AI agent for psychotherapy. Hypothesis 1 is then accepted with higher responses of low-to-moderate levels of awareness and trust in using AI in mental health support.

### 3.2. Readiness and Preference

To address Hypothesis 2, we used the readiness question: “Are you willing to use AI applications to manage stress?” The responses were 18.2% “no,” 56.3% “yes,” and 25.5% “I don’t know.” The one-way Chi-Square analysis showed a significant difference with χ^2^(2, *n* = 220) = 44.4, *p* < 0.0001. Suggesting that pupils are now ready to use AI. In terms of preference, the question: “Would you prefer using AI applications for mental health instead of traditional face-to-face counseling?” Students answered: 56.4% “no,” 21.8% “yes,” and 21.8% “don’t know.” The Chi-Square test showed significance between the categories χ^2^(2, *n* = 220) = 52.51, *p* < 0.0001. Students were less likely not to use AI applications to seek counseling, and thus Hypothesis 2 is rejected.

### 3.3. Readiness and Expectations

To address Hypothesis 3, we crossed the factor of readiness with expectation. A cross-table was created to determine whether students are ready to use AI. We used the question: “Are you willing to use AI applications to manage stress?” crossed by expectations: “Do you think AI can help provide initial psychological support to students?” As reported above, the majority responded they were willing to use AI with a significant Chi-Square (*p* < 0.0001). A tabulated readiness against expectations showed a strong association between the two variables χ^2^(4, *n* = 220) = 35.87, *p* < 0.000. Those who showed preference were not sure they were ready. Likewise, those who were ready to use AI did not expect it to provide initial psychological support (see Table 4). Thus, supporting Hypothesis 3.

### 3.4. Gender and Academic Level on Awareness, Trust, Readiness, and Preferences

To address differences between male and female, and undergraduate and graduate student responses on the four factors awareness, trust, readiness, and preferences, we recoded all the item responses from 1 = “No”, 2 = “I don’t know”, and 3 = “Yes” as a scale from lower to higher rating, respectively. A one-way ANOVA was used for each of the questions that addressed each factor. We performed Lavene’s test to address the homogeneity of variance of the independent factors on awareness, trust, readiness, and preferences. The assumption was satisfied with the exception of gender preference with a Lavene’s F (1, 218) = 13.36, *p* = 0.000. Table 5 presents the results of the analysis. There was no significant difference between males and females and between graduate and undergraduate students across all the factors of awareness, trust, readiness, and preferences. The effect size in the analyses showed minimal and significant effects, suggesting that gender and education level do not substantially influence the factors of awareness, trust, readiness, and preferences. Even though the gender on preference assumption of equal variance was not fulfilled, the effect size was extremely small. Therefore, the insignificant difference between males and females in their preferences should not be a concern, as the practical impact of this variance is minimal. Thus, Hypothesis 4 was accepted as males and females, undergraduates and graduates have the same percentage of responses.

To address Research Question 4 of this study, we descriptively explored the concerns and benefits regarding the incorporation of AI (see Table 6). The concern construct where two questions were used: “What are your biggest concerns about using AI in mental health? And, “Do you agree with the use of artificial intelligence in diagnosing mental disorders?” The most prolific response to concern was the loss of human contact (34.59%), followed by overreliance on technology (27.57%). On the second question, pupils were concerned about the actual diagnostic accuracy (41.18%), followed by prescribing the correct treatment (29.08%). In terms of benefits, pupils with the highest ranked response saw treatment costs reduced significantly (35.2%), followed by traditional face-to-face, as well as providing 24/7 (27.75%).

## 4. Discussion

This study investigated university students’ perceptions of AI in supporting mental health. Students’ perceptions include awareness, trust, readiness, preferences, expectations, and perceived concerns and benefits of employing AI in mental health. The following discussion is organized in accordance with the research questions and hypotheses to provide a coherent interpretation of the findings.

Consistent with Research Question 1, the findings indicate that students demonstrated low-to-moderate levels of awareness and trust in employing AI in mental health. This supports Hypothesis 1, which predicted limited awareness and trust. The results showed intermediate trust levels in employing AI in mental health, indicating that students remain cautious in relying on AI for emotionally complex issues. This finding aligns with the existing digital mental health literature that focuses on using AI technologies to offer accessibility and innovation. However, using AI technologies in mental health is often constrained by concerns regarding accuracy, confidentiality, and emotional understanding [39,75]. Trust in employing AI in mental health is shaped by both technical performance (e.g., reliability and diagnostic precision) and affective components (e.g., empathy and emotional safety) [76,77]. Thus, students’ cautious trust reflects a balanced evaluation rather than a rejection of employing AI in mental health, which is consistent with recent findings emphasizing critical engagement with AI technologies [4].

In relation to Research Question 2, the results showed that while the majority of students reported readiness to use AI for stress management, they expressed a low preference for replacing traditional face-to-face psychotherapy with AI tools. As a result, Hypothesis 2 was not supported. This finding suggests that students differentiate between the functional use of AI (e.g., stress management, self-help tools) and relational therapeutic contexts, where human interaction and empathy remain essential. Our results are in line with recent studies that showed that AI is more acceptable for supplementary roles such as screening, monitoring, and psychoeducation but less acceptable as a standalone therapeutic substitute [78,79]. In addition, these findings support the growing consensus that AI in mental health is best positioned within hybrid care models, where digital tools enhance accessibility while human therapists provide depth, empathy, and unconditional acceptance [80,81].

Hypothesis 3, the relationship between “readiness” and “expectations” of employing AI in mental health, was examined. The results showed a significant association between readiness and expectations, supporting Hypothesis 3. However, this relationship was not linear or straightforward. Interestingly, some students who expressed readiness to use AI did not necessarily believe that AI could provide initial psychological support, while others who perceived AI as useful, were uncertain about their readiness to engage with it. This uncertainty reflects a cognitive–behavioral dissonance where perceived usefulness does not always translate into behavior. Such findings are consistent with technology acceptance research, which suggests that adoption is influenced not only by perceived benefits but also by emotional trust, perceived risks, and contextual factors [22,82]. This highlights the importance of addressing both cognitive expectations and emotional concerns in employing AI in mental health.

Regarding Research Question 3, the results showed no significant differences in awareness, trust, readiness, or preferences based on gender or academic level. Therefore, Hypothesis 4 was supported. Our results may be explained by shared generational exposure to digital technologies, resulting in relatively homogeneous attitudes toward AI among university students. Our findings align with previous studies showing that digital-native populations exhibit comparable engagement with AI across demographic groups [83,84,85,86,87]. However, this contrasts with some Western studies reporting gender-based differences in AI use [88]. This contrast in data suggests that cultural and contextual factors may moderate such variations [89].

In addressing Research Question 4, the findings revealed that students’ perceptions of AI in mental health are shaped by a balance between perceived benefits and concerns. The most prominent concerns included loss of human interaction, overdependence on technology, diagnostic accuracy, privacy, and data security. These concerns echo prior research emphasizing the importance of ethical safeguards, transparency, and accountability in AI-driven mental health systems [90,91]. At the same time, students recognized several key benefits, including 24/7 availability, reduced costs, and increased accessibility, which are consistent with previous findings on the advantages of digital mental health interventions [92]. This dual perception highlights a central concern in AI adoption that while using AI in counseling enhances efficiency and accessibility, it may compromise emotional depth, empathy, and interpersonal connection, which remain fundamental to mental health care [93,94]. Our findings should be interpreted within the cultural context of the study. Evidence from Arab and Gulf societies suggests that help-seeking behaviors are influenced by stigma, social visibility, and privacy concerns [75,77,95,96], and hence, students are less likely to declare their mental health issues compared to Western counterparts, and the use of face-to-face therapy may be too embarrassing and revealing. In line with the literature, students emphasized anonymity and confidentiality as key advantages of AI-based tools, which may help reduce the stigma associated with mental health disclosure. Previous studies have similarly shown that AI can provide private and less socially visible support options [41,97,98]. However, concerns regarding personal data security and institutional access to personal information remain significant barriers [84,99], reflecting broader global patterns in privacy-related apprehensions [100,101].

Overall, our findings suggest that students adopt a cautious but open stance toward employing AI in mental health. Acceptance is influenced by a combination of trust, perceived usefulness, cultural factors, and ethical concerns, rather than purely technological factors. Importantly, students do not view AI as a replacement for human therapy but rather as a complementary tool that can enhance accessibility, provide initial support, and assist in monitoring mental health. This reinforces the importance of developing ethically governed, culturally sensitive, and hybrid mental health care models that integrate AI while preserving the central role of human interaction.

### Limitation

This study has several limitations that should be considered when interpreting the findings. First, the use of voluntary participation introduces the possibility of self-selection bias, as students who chose to participate may have had a greater interest in mental health or AI technologies. This may have resulted in a sample that is not fully representative of the broader university population and could have influenced the overall pattern of responses. Second, the sample was predominantly composed of students from counseling and mental health programs and was largely female, which may further limit the generalizability of the findings to other academic disciplines or more gender-balanced populations. Third, a cross-sectional design restricts the ability to establish causal relationships or examine changes in perceptions over time. Fourth, although non-parametric and robust statistical analyses were employed, these methods do not address underlying sampling bias and therefore do not eliminate concerns related to representativeness. Fifth, this study was exploratory, and single-item measures were deliberately employed to capture these dimensions in a concentrated manner. Single-item indicators restrict the capacity for reliability or factor-analytic validation; however, their utilization does not diminish the overall interpretation of the results. In exploratory research, single items can efficiently assess general perceptions, particularly when constructs are tangible, easily comprehensible, and not anticipated to encompass multidimensional frameworks. Consequently, the dependence on single-item measures should be regarded as a methodological decision aligned with the objectives of this preliminary study, and it does not undermine the significance or interpretive utility of the identified patterns in the data. Finally, the findings should be interpreted within the specific cultural and institutional context of a single university in Qatar. As such, the results are best understood as context-specific and exploratory, and caution is warranted in generalizing them to other populations or settings. Future research should employ more diverse, multi-institutional, and probabilistic sampling strategies to enhance external validity.

## 5. Conclusions

Findings from this study showed the perspectives of university students in the Arab Gulf on AI in mental health. The findings showed that trust plays a significant role in determining their readiness to adopt such technologies. Students showed a cautious willingness to employ AI in mental health as they had concerns about privacy, empathy, diagnostic accuracy, and emotional interaction. The students did not see AI as a substitute for human therapy, despite their recognition of AI’s possible advantages, including accessibility and ease.

These findings reflect students’ perceptions rather than actual usage or effectiveness of AI-based interventions. Accordingly, AI-based tools may be considered as potential complementary resources, as perceived by students, rather than established solutions within mental health services. Given the cross-sectional design and single-institution sample, the findings should be interpreted with caution, and further research is needed to evaluate the effectiveness, implementation, and broader applicability of AI in mental health care across diverse contexts.

## Figures and Tables

**Table 1:** Dimensions, questions, and responses.

Dimension	Instrument Question	Response
Awareness	What is the extent your knowledge of AI applications in the field of mental health?	Weak, Moderate, Good, Excellent
Trust	How confident are you in using AI for psychotherapy?	Low, Good, Intermediate
Readiness	Are you willing to use AI applications to manage stress?	No, Yes, I Don’t Know
Preference	Would you prefer using AI applications for mental health instead of traditional face-to-face counseling?	No, Yes, I Don’t Know
Expectations	Do you think AI can help provide initial psychological support to students?”	No, Yes, I Don’t Know
Concern	What are your biggest concerns about using AI in mental health?	-Privacy and data security -Diagnostic accuracy -Loss of human communication -Overdependence on technology -Other -Strongly disagree -Disagree -Neutral -Agree -Strongly agree
	Do you agree with the use of artificial intelligence in diagnosing mental disorders?	
Benefit	What benefits do you expect from using AI to treat psychological disorders?	-Speed of diagnosis -Providing support 24/7 -Reducing treatment costs -Improving diagnostic accuracy -Increasing access to mental-health care Other

**Table 2:** Expert validation of the research instrument.

Expert No.	Expertise/Academic Title	Institutional Affiliation	Years of Experience	Focus Area of Review	Key Feedback/Modifications Implemented
1	Professor of Clinical Psychology	Damascus University	20	Concept clarity, cultural alignment	Suggested simplifying terms related to “AI psychotherapy” to improve comprehension for non-psychology students.
2	Associate Professor of Counseling Psychology	British University in Egypt	15	Content coverage, construct representation	Recommended adding separate items for “trust” and “perceived usefulness.”
3	Assistant Professor of Mental Health	Future University, Egypt	10	Relevance and item clarity	Proposed reordering sections to improve logical flow from awareness to readiness.
4	Psychiatrist (Mental Health Specialist)	Ministry of Public Health, Qatar	9	Cultural sensitivity, ethical framing	Advised modifying language around “mental disorders” to “psychological difficulties” for inclusivity.
5	Professor of Clinical Neuropsychology	University of Aleppo	25	Measurement consistency	Suggested converting open-ended questions into Likert-scale items to enhance statistical analysis.
6	Assistant Professor of Mental Health Psychology	Lusail University	8	Digital context validity	Recommended integrating “readiness to use AI” scale items to align with technology acceptance frameworks.
7	Clinical Supervisor/Counseling Specialist	Qatar University	18	Practical applicability	Endorsed inclusion of a question on privacy and ethical concerns regarding AI tools.
8	Researcher in AI and Mental Health	Independent Consultant	10	Technical accuracy	Recommended specifying examples of AI tools (e.g., chatbots, apps) to ensure consistent interpretation among participants.

**Table 3:** Demographics of the sample.

		*n*	Percentage	Minimum	Maximum	Mean	Standard Deviation
	Age	220	100	15	55	27.00	7.639
Gender	Male	48	21.8				
	Female	172	78.2				
Major of Study	Counseling and Mental Health	145	65.9				
	Law	22	10.0				
	Information Systems	23	10.5				
	English Language Teaching	4	1.8				
	Business Administration	26	11.8				

**Table 4:** A 3 × 3 contingency table of readiness by expectations.

Readiness: How Willing Are You to Use AI Applications to Help Manage Stress?					
		Yes	No	I don’t know	Total
Expectations: Do you think AI can help provide initial psychological support to students?	Yes	6	24	74	104
	No	25	13	25	63
	I don’t know	9	19	25	53
Total		40	56	124	220

χ^2^_cr_ @ α = 0.01 is 13.28.

**Table 5:** Gender and academic level by trust, readiness, awareness, and preferences.

	Male Mean (SD)	Female Mean (SD)	F (df)	Effect Size (η^2^)	Undergrad. Mean (SD)	Post-Graduate Mean (SD)	F (df)	Effect Size (η^2^)
Awareness								
How familiar are you with AI applications in mental health?	1.90 (0.75) 2	1.81 (0.69)	0.51 (1218)	0.0023	1.83 (0.71)	1.94 (0.68)	0.36 (1217)	0.0017
Trust								
How confident are you in using AI for psychotherapy?	1.75 (0.57)	1.62 (0.65)	1.53 (1218)	0.007	1.64 (0.63)	1.88 (0.72)	2.13 (1217)	0.010
Readiness								
Are you willing to use AI applications to manage stress?	2.46 (0.71)	2.36 (0.79)	0.60(1218)	0.0027	2.36 (0.78)	2.69 (0.60)	2.6 (1217)	0.0118
Would you use AI-powered chatbots for initial psychological support?	1.88 (0.82)	1.71 (0.77)	1.69 (1218)	0.0077	1.76 (0.78)	1.56 (0.814)	0.98 (1217)	0.0045
Preferences								
Would you prefer using AI applications for mental health instead of traditional face-to-face counseling?	1.79 (0.94)	1.62 (0.78)	1.74 (1218)	0.0079	1.67 (0.82)	1.56 (0.73)	0.23 (1217)	0.0011

**Table 6:** Descriptive information about concerns and benefits.

Variable	Category/Response	Frequency	Percent	Rank
Concerns	Loss of human contact	138	34.59%	1
	Overreliance on technology	110	27.57%	2
	Diagnostic accuracy	88	22.06%	3
	Privacy and data security	60	15.04%	4
	Other concerns	3	0.75%	5
Concerns: Challenges	Diagnostic accuracy	126	41.18%	1
	Prescribing correct treatment	89	29.08%	2
	Difficulty of access	55	17.97%	3
	Stigma	20	6.54%	4
	Other challenges	16	5.23%	5
Benefits	Reducing treatment costs	137	35.22%	1
	Providing 24/7 support	108	27.76%	2
	Speed of diagnosis	78	20.05%	3
	Increasing access to mental-health care	59	15.17%	4
	Diagnostic accuracy	7	1.80%	5

## Data Availability

The corresponding author can provide reasonable access to the datasets created during and/or analyzed during the current investigation.
